# Three-year reliability of MEG resting-state oscillatory power

**DOI:** 10.1016/j.neuroimage.2021.118516

**Published:** 2021-08-25

**Authors:** Brandon J. Lew, Emily E. Fitzgerald, Lauren R. Ott, Samantha H. Penhale, Tony W. Wilson

**Affiliations:** a Institute for Human Neuroscience, Boys Town National Research Hospital, Boys Town, NE, USA; b College of Medicine, University of Nebraska Medical Center, Omaha, NE, USA

**Keywords:** Magnetoencephalography, Resting-State, ICC, Oscillations, Stability, Test-retest

## Abstract

**Introduction:**

Resting-state oscillatory activity has been extensively studied across a wide array of disorders. Establishing which spectrally- and spatially-specific oscillatory components exhibit test-retest reliability is essential to move the field forward. While studies have shown short-term reliability of MEG resting-state activity, no studies have examined test-retest reliability across an extended period of time to establish the stability of these signals which is critical for reproducibility.

**Methods:**

We examined 18 healthy adults age 23 – 61 who completed three visits across three years. For each visit participants completed both a resting state MEG and structural MRI scan. MEG data were source imaged, and the cortical power in canonical frequency bands (delta, theta, alpha, beta, low gamma, high gamma) was computed Intra-class correlation coefficients (ICC) were then calculated across the cortex for each frequency band.

**Results:**

Over three years, power in the alpha and beta bands displayed the highest reliability estimates, while gamma showed the lowest estimates of three-year reliability. Spatially, delta, alpha, and beta all showed the highest degrees of reliability in the parietal cortex. Interestingly, the peak signal for each of these frequency bands was located outside of the parietal cortex, suggesting that reliability estimates were not solely dependent on the signal-to-noise ratio.

**Conclusion:**

Oscillatory resting-state power in parietal delta, posterior beta, and alpha across most of the cortex are reliable across three years and future MEEG studies may focus on these measures for the development of specific markers.

## Introduction

Neuroimaging of resting-state activity has become widely popular, and as a result, our understanding of systems-level neural function has dramatically expanded. Many studies have begun to extend this knowledge to examine resting-state activity in neurologic and psychiatric disorders (Newson and Thiagarajan, 2019) to identify disease biomarkers. Clinically, the utilization of resting-state paradigms is ideal, given its ease of acquisition compared to a task-based paradigm, which can pose performance challenges due to cognitive status and other factors. However, for such biomarkers to be scientifically reproducible and clinically useful, the metrics obtained through resting-state functional neuroimaging must be reliable from scan to scan. Essentially, it is well accepted that severe psychiatric and neurological disorders are persistent and often lifelong conditions, and consequently a marker of such conditions should exhibit the same characteristics. Furthermore, for such a marker to be useful in the identification/diagnosis of disease, its variability must be within a range that enables the patient and control distributions on the measure of interest to be distinguished. Long-term stability also enables measures to be used as screening tools in healthy populations, as an alteration in the measure beyond what would be expected from increasing age can then be interpreted as indicative of emerging pathology. In short, to be useful as a marker of disease or cognitive construct, it is important that such measurements accurately capture stable traits of the participants and not states which are likely to be more variable. Identifying such robust and reproducible markers is essential for the advancement of MEEG research.

Test-retest reliability in functional neuroimaging remains a timely and important topic across multiple modalities, including fMRI, EEG, and MEG (Elliott et al., 2020; McCusker et al., 2020; McEvoy et al., 2000). As defined by Elliot et al. (2020), test-retest reliability refers to the reproducibility of results from a given measure under nearly equivalent conditions. A common statistic used to assess test-retest reliability is the intraclass correlation coefficient (ICC), which is typically defined the ratio of the variance of interest over the sum of the variance of interest plus error (Bartko, 1966; Ebel, 1951; Shrout and Fleiss, 1979). According to Koo & Li (2016), ICC values less than .5 indicate poor reliability, values between .5 and .75 indicate moderate reliability, values between .75 and .9 indicate good reliability, and values greater than .9 indicate excellent reliability. Despite multiple studies examining the reliability of functional neuroimaging techniques, the stability across various years of these measures is still widely debated in the literature (Bennett and Miller, 2010; Elliott et al., 2020; Kragel et al., 2020; Lieberman et al., 2009; Vul et al., 2009). Thus, additional work is necessary to distinguish which neuroimaging metrics are more or less reliable, and to determine whether these estimates are stable over more extended time periods, which is the goal of the current study.

While most resting-state functional neuroimaging work has been performed with fMRI, resting-state MEG and EEG offer unique insight into the underlying oscillatory activity. Such oscillatory activity is important in cognition (Başar et al., 2001) and aberrant in many disorders (Bosboom et al., 2006; Engels et al., 2017; Uhlhaas and Singer, 2006; Zeev-Wolf et al., 2018). Previous studies have examined short-term test-retest reliability of resting-state oscillatory activity using EEG (Fingelkurts et al., 2006; Gasser et al., 1985; Gudmundsson et al., 2007; McEvoy et al., 2000; Salinsky et al., 1991), and found high reliability estimates for multiple alpha-frequency measures. These studies were conducted at the scalp level, which has limited insight into whether the underlying brain regions generating the alpha activity remain the same across time. A smaller number of MEG studies have examined test-retest reliability of resting-state metrics. Notably, Martín-Buro et al. (2016) investigated the short-term reliability of resting-state power spectra in healthy adults using MEG and found high reliability estimates for theta, alpha, and low beta power. Other studies of the reliability of MEG measures have explicitly looked into resting-state network connectivity and task-based responses (Colclough et al., 2016; Garcés et al., 2016; H.-R. M. Tan et al., 2016).

Beyond these short-term investigations of test-retest reliability, EEG studies that have investigated long-term intra-individual reliability have found spectral parameters (Näpflin et al., 2007), task-based neural oscillations (Neuper et al., 2005), quantitative EEG more broadly (Kondacs and Szabó, 1999), and sleep metrics to have relatively good reliability (Perkinson-Gloor et al., 2015). In contrast, studies of long-term reliability using MEG have not been extensively performed. The few studies of long-term test-retest reliability that have utilized MEG focused on visual and somatosensory task-based responses (McCusker et al., 2020), and a resting state study examining patients with HIV at the MEG sensor level (Becker et al., 2012). Both studies suggested moderate to high reliability for most MEG metrics, but obviously further work is needed. Given the growing trend of using MEG to derive biomarkers of disease and treatment response (Wilson et al., 2016), it will be critical to determine the long-term test-retest reliability of common MEG metrics of neural oscillatory activity.

In the current study, we investigated the long-term test-retest reliability of MEG-derived resting-state oscillatory power measures in a group of healthy adults who completed three separate visits equally spaced across 36 months. Using this longitudinal design, we calculated the ICC as a measure of the long-term test-retest reliability of frequency-specific and spatially-specific responses in anatomical space. Thus, our ICC measures of *reliability* reflect an estimate of the *stability* of regional oscillatory power within each frequency band over time (Jin et al., 2011). Our primary hypotheses were that the frequency bands and regions with the strongest power (e.g., occipital alpha) would have good to excellent reliability across the three years. Frequency bands and regions with less power would generally have poorer reliability.

## Methods

### Participants

We enrolled 18 healthy adults (11 Male, 7 Female; 16 right-handed, 2 left-handed) who participated in three separate visits (Visit 1: *M*_age_ = 44.68 years, range = 23.9 – 61.9 years; Visit 2: *M*_age_ = 46.27 years, range = 25.5 – 63.5; Visit 3: *M*_age_ = 47.01 years, range = 27.0 – 65.1). The average time between Visit 1 and Visit 2 was 1.60 years (range = 1.45 – 1.70 years), while the average time between Visit 2 and Visit 3 was 1.51 years (range = 1.34 – 1.66 years) for a total average time of 3.11 years (range = 3.02 – 3.30 years) between Visits 1 and 3. Exclusionary criteria included any medical illness affecting CNS function, neurological or psychiatric disorder, history of head trauma, current substance misuse, and non-removable metal implants that would adversely affect data acquisition. Each participant provided written informed consent and was compensated for their time and travel. The Institutional Review Board at the University of Nebraska Medical Center reviewed and approved this study, and all protocols were in accordance with the Declaration of Helsinki.

### MEG data acquisition

All MEG recordings took place in a one-layer magnetically-shielded room with active shielding engaged for environmental noise compensation. A 306-sensor Elekta/MEGIN MEG system (Helsinki, Finland), equipped with 204 planar gradiometers and 102 magnetometers, was used to sample neuromagnetic responses continuously at 1 kHz with an acquisition bandwidth of 0.1 – 330 Hz. The same instrument was used across all recordings. Participants were seated in a custom-made non-magnetic chair within a magnetically shielded room, with their heads positioned within the sensor array. Participants were instructed to rest with their eyes closed for 6 min and were monitored by a real-time audio-video feed from inside the shielded room throughout MEG data acquisition.

### Structural MRI acquisition, processing, and MEG-MRI coregistration

Individual structural MRI data were obtained from all participants and at all three visits. All T1-weighted sMRI data were acquired with a Philips Achieva 3T X-series scanner using an 8-channel head coil (TR: 8.09 ms; TE: 3.7 ms; field of view: 240 mm; slice thickness: 1 mm; no gap; in-plane resolution: 1.0 × 1.0 mm). Participants’ high-resolution T1-weighted MRI data were segmented using the standard voxel-based morphometry pipeline in the computational anatomy toolbox (CAT12 v12.6; Gaser and Dahnke, 2016) within SPM12. Repeated measurements of the MRI data (three visits) were accounted for by utilizing CAT12’s longitudinal segmentation model, which first utilizes a symmetric realignment and an intra-subject bias field correction (Ashburner and Ridgway, 2013; Reuter et al., 2010; Reuter and Fischl, 2011). These resulting images then underwent noise reduction using a spatially-adaptive non-local means denoising filter (Manjón et al., 2010) and a classical Markov Random Field approach (Rajapakse et al., 1997). An affine registration and a local intensity transformation were then applied to the bias-corrected images. Finally, preprocessed images were segmented based on an adaptive maximum a posterior technique (Ashburner and Friston, 2005) and a partial volume estimation with a simplified mixed model of a maximum of two tissue types (Tohka et al., 2004). Images were normalized to MNI template space. The resulting segmented files were then imported into Brainstorm for coregistration.

Prior to MEG acquisition, four coils were attached to the participants’ heads and localized, together with the three fiducial points and scalp surface, using a 3-D digitizer (Fastrak 3SF0002, Polhemus Navigator Sciences, Colchester, VT, USA). Once the participant was positioned for MEG recording, an electrical current with a unique frequency label (e.g., 322 Hz) was fed to each coil. This induced a measurable magnetic field and allowed each coil to be localized in reference to the sensors throughout the recording session. Since coil locations were also known in head coordinates, all MEG measurements could be transformed into a common coordinate system. With this coordinate system (including the scalp surface points), each participant’s MEG data were co-registered with the structural magnetic resonance images (sMRI) prior to source space analyses using Brainstorm.

### MEG data pre-processing

Each MEG dataset was individually corrected for head motion and subjected to noise reduction using the signal space separation method with a temporal extension (tSSS; MaxFilter v2.2; correlation limit: 0.950; correlation window duration: 6 seconds; Taulu and Simola, 2006). MEG data processing then largely followed the same analysis pipeline outlined in (Niso et al., 2019). Noise-reduced MEG data underwent standard data preprocessing procedures using the Brainstorm software (Tadel et al., 2011). A high pass filter of 0.3 Hz and notch filters at 60 Hz and at its harmonics were applied. Cardiac artifacts were identified in the raw MEG data and removed using an adaptive signal-space projection (SSP) approach, which was subsequently accounted for during source reconstruction (Ille et al., 2002; Uusitalo and Ilmoniemi, 1997). Data were then divided into four-second epochs for detection and rejection of bad segments of data. Amplitude and gradient metrics for each epoch were computed, and epochs containing outlier values were rejected using an individualized fixed threshold method, supplemented with visual inspection (Visit 1 *M*epochs = 79.667, Visit 2 *M*epochs = 79.889, Visit 3 *M*epochs = 82.722). Repeated measures ANOVA showed that the number of epochs did not significantly differ across visits (F(2, 34) = 1.535, p = 0.230). Briefly, in MEG, the raw signal amplitude is strongly affected by the distance between the brain and the MEG sensor array, as the magnetic field strength falls off sharply as the distance from the current source increases. To account for this source of variance across participants and other sources of variance, we used an individually determined threshold based on the signal distribution for both amplitude and gradient to reject artifacts.

To ensure the reproducibility of our preprocessing procedures, the identification of cardiac artifacts, determination of individualized thresholds for epoch rejection, and data coregistration were performed by three independent raters. Each of the three datasets were then fully processed to the final outcome metrics, and then inter-rater reliability was assessed. (see Supplement: Fig. S1)

### MEG source imaging and frequency power maps

As in (Niso et al., 2019), we then computed minimum norm estimates normalized by a dynamic statistical parametric mapping (dSPM) algorithm for source imaging. To account for environmental noise, we utilized empty room data to compute a noise covariance matrix for source imaging (Baillet et al., 2001). The forward model was computed using an overlapping spheres head model (Huang et al., 1999) with 15000 cortical surface vertices. Finally, the imaging kernel of depth-weighted dynamic statistical parametric mapping (dSPM) constrained to the individual cortical surface (Dale et al., 2000) was computed.

Using these source estimates, we then estimated the power of cortical activity in the canonical frequency bands: delta (2–4 Hz), theta (4–8 Hz), alpha (8–12 Hz), beta (15–30 Hz), low gamma (30–80 Hz), and high gamma (80–150 Hz). We used Welch’s method for estimating power spectrum densities (PSD) per four-second epoch across each MEG recording, with 1-second sliding Hamming windows overlapping at 50%. We then standardized the PSD values at each frequency bin to the total power across the frequency spectrum. We then averaged PSD maps (ie. source estimates) across epochs for each participant to obtain one set of six PSD maps (one per frequency band) per participant per visit. Finally, we projected these maps onto the MNI ICBM152 brain template (Fonov et al., 2009) and applied a 3 mm FWHM smoothing kernel. Ultimately, it is these normalized source maps per frequency band that were used for further statistical analysis.

### Statistical analyses

We first computed grand-averaged PSD maps, averaged across all participants and all visits. Next, we calculated intraclass correlation coefficients (ICC) to measure the extent of source-level reliability among each frequency band across the three visits. We only investigated the source level maps because previous MEG studies have found that the reliability of sensor and estimated source signals are comparable (H.-R. Tan et al., 2016; Tan et al., 2015), with source space having greater reliability due to several advantages (Martín-Buro et al., 2016; H.-R. Tan et al., 2016). Specifically, we implemented a single rater two-way mixed-effects model and absolute agreement definition, or ICC(A,1) defined by McGraw and Wong (1996) and based on Shrout and Fleiss (1979). This ICC definition is generally more conservative, and additionally, Koo and Li (2016) suggest using a two-way mixed-effects model and absolute agreement definition for test-retest reliability studies, which coincides with our long-term reliability design. ICC estimates and their 95% confidence intervals were calculated using the Matlab Central file-exchange *ICC.m* function (Salarian, 2016) in Matlab (Version 2018b; Mathworks, Inc., Massachusetts, USA). This ICC calculation was applied at every vertex in the PSD maps to obtain spatially specific reliability estimates at each of the frequency bands. This resulted in an ICC map per frequency band.

To further visualize the reliability of source power in each frequency band, regions of interest (Brainstorm “scouts”) in the frontal, parietal, temporal, and occipital lobes were applied to each participant’s PSD map. The average power (relative to total spectral power) across each lobe was extracted for each participant and each visit. ICCs of these values were then calculated using the same ICC(A,1) model.

For a brief background, ICC estimates range from 0 – 1, with values closer to 1 indicating higher reliability. Low ICCs could be due to several reasons: 1) a low degree of rater (i.e., visit) or measurement agreement, 2) a lack of variability in sample participants, 3) a small number of participants, and 4) a small number of raters (i.e., visits) being tested (Chen et al., 2017; Koo and Li, 2016). Interpretations of ICC estimate values vary (Cicchetti, 1994; Koo and Li, 2016; Portney and Watkins, 2009), but we followed a general consensus among these sources with values less than .5 indicating poor reliability, values between .5 and .75 indicating moderate reliability, values between .75 and .9 indicating good reliability, and values greater than .9 indicating excellent reliability. ICCs of .7 or higher are usually considered necessary to study individual psychometric or behavioral differences (Boutros et al., 1991; Kline, 2000). Importantly, we evaluated the level of reliability based on the 95% confidence interval of the ICC estimate, not the estimate itself, since the interval reveals the chance that the true ICC value lands on any point between the bounds.

## Results

### MEG source mapping results

Overall, grand averaged source PSD maps showed highly similar power distributions reported in previous literature (Niso et al., 2019, 2016). ICC estimates of reliability over three years were then calculated on these source PSD maps. Generally, alpha and beta frequency bands showed the highest estimates of reliability. Spatially, the parietal cortex displayed the highest degree of reliability in multiple frequencies, including delta, alpha, and beta. Below we discuss the results in each frequency band in more detail.

### Delta

**Fig. 1.**
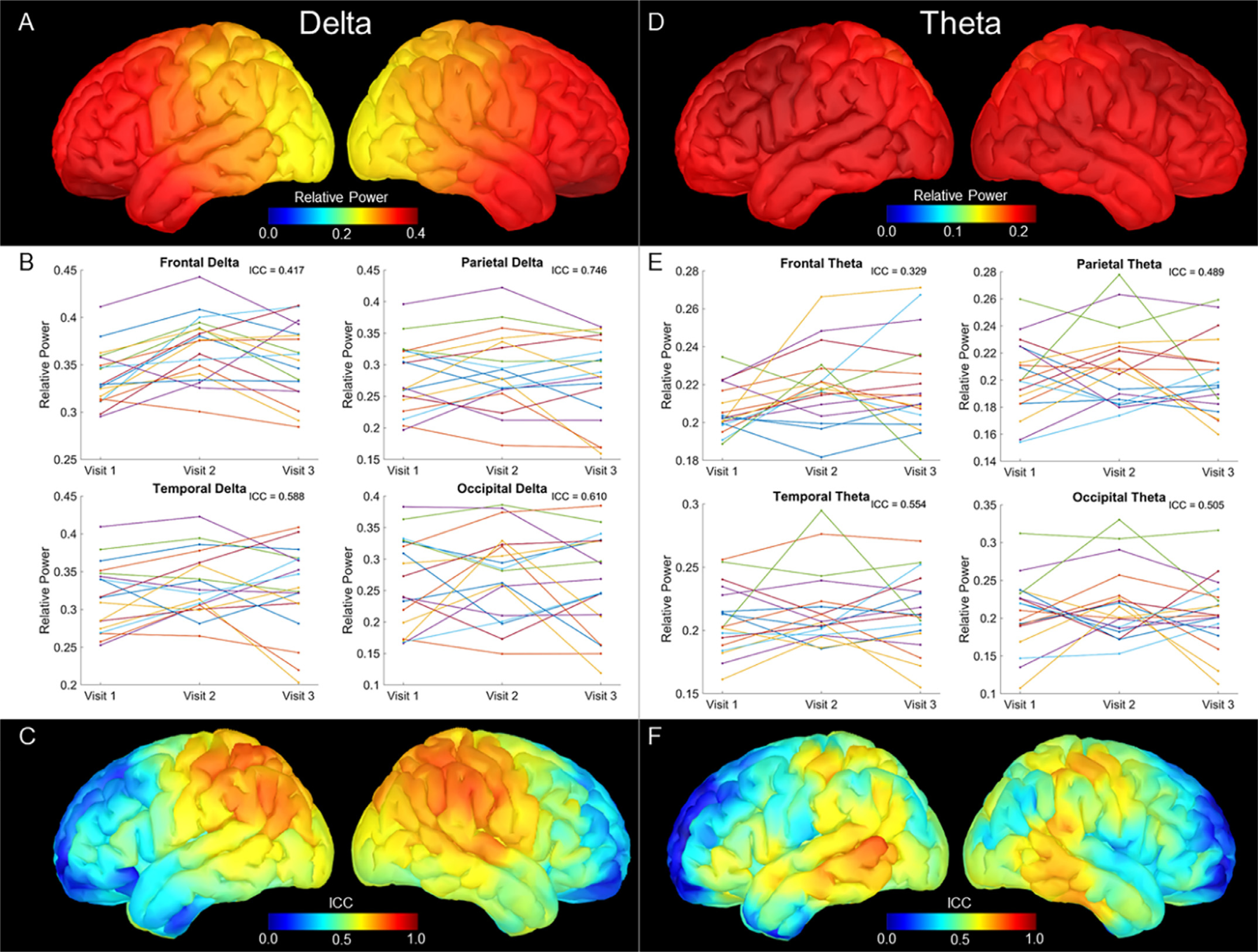
Delta and Theta Relative Power Estimations and 3-year ICC Estimates. Top panels show grand averaged relative power maps, with color bars scaled to the maximum values of the map. Delta (A) showed predominantly frontal power, and theta (D) showed an even distribution of power. Middle panels display relative power extracted for each participant, with one plot for each lobe. Each line represents a participant, with visit on the x axis and the average relative power across each lobe on the y axis. ICC estimates are inset and reveal the highest estimates were in the parietal lobe for delta (B), and the temporal lobe for theta (D). Bottom panels display vertex-wise ICC estimates for the three visits, scaled from 0 to 1. Delta (C) reliability spans the parietal lobe, while theta (F) reliability is relatively sparse.

Grand average maps of resting delta (2–4 Hz) power showed the highest power in the orbitofrontal cortex and generally decreased in power posteriorly (Fig. 1A). ICC estimates at each of the lobes showed the highest reliability in the parietal cortex (ICC = .746, 95% CI: .539-.885), followed by the occipital lobe (ICC = .610, 95% CI: .349-.813), temporal lobe (ICC = .588, 95% CI: .324-.800), and the lowest reliability in the frontal lobe (ICC = .417, 95% CI: .134-.690; Fig. 1B). Vertex-wise ICC maps show a moderate level of reliability across the parietal cortex, generally decreasing ICC values with distance from the parietal lobe (Fig. 1C). Notably, delta power in the orbitofrontal cortex showed poor reliability, with ICC estimates approaching zero.

### Theta

Grand average maps of resting theta (4–8 Hz) power showed broadly even power across the cortex, with the highest power in the dorsomedial prefrontal cortex (Fig. 1D). ICC estimates at each of the lobes displayed broadly poor reliability, with the highest estimates in the temporal cortex (ICC = .554, 95% CI: .281-.780), followed by the occipital lobe (ICC = .505, 95% CI: .224-.750), parietal lobe (ICC = .489, 95% CI: .207-.740), and the lowest reliability in the frontal lobe (ICC = .329, 95% CI: .063-.621; Fig. 1E). Vertex-wise ICC maps show a small spatial cluster of moderate reliability in the middle temporal gyrus (Fig. 1F). However, overall, ICC estimates were quite modest, with most of the cortex below 0.5 (poor reliability).

### Alpha

**Fig. 2.**
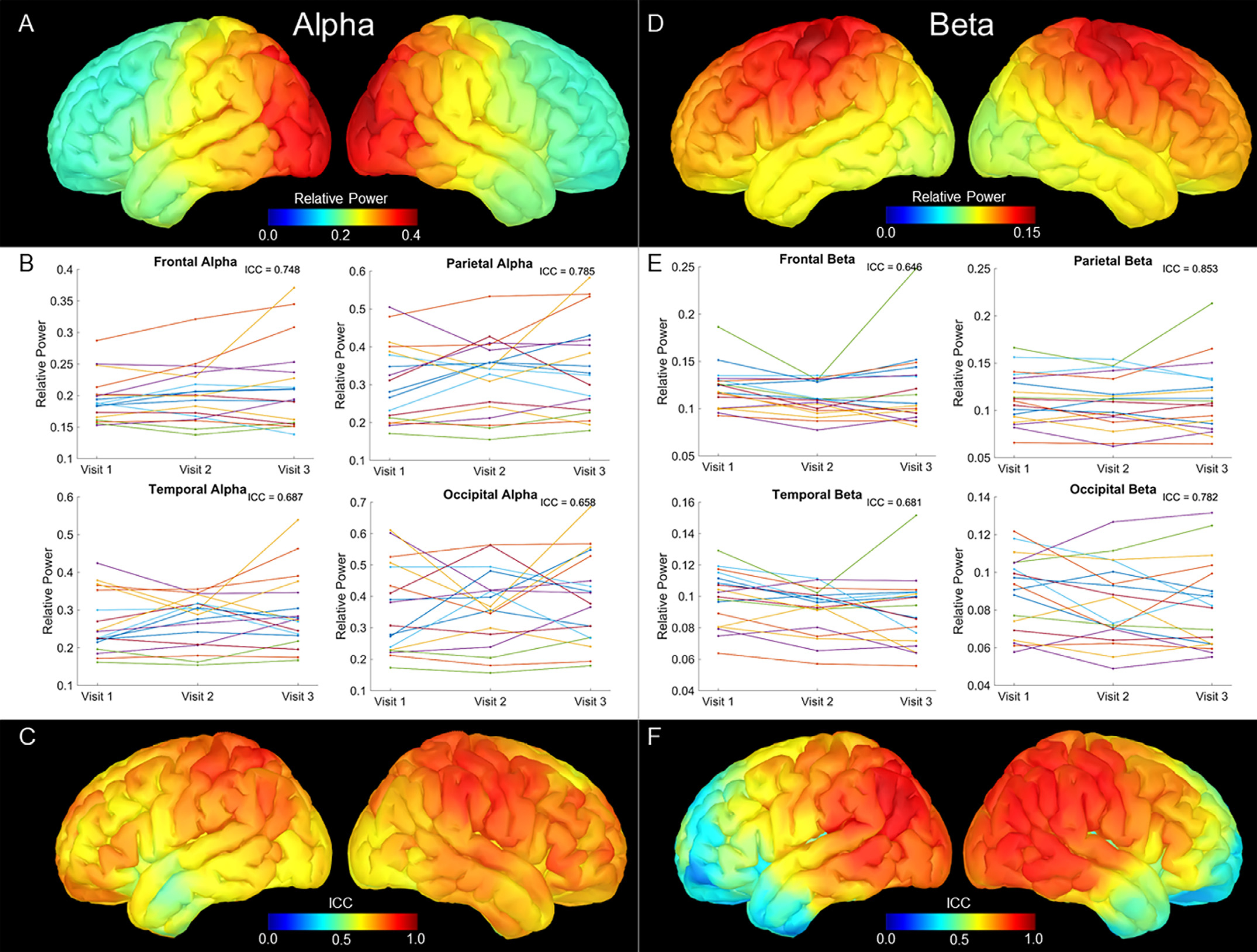
Alpha and Beta Relative Power Estimations and 3-year ICC Estimates. Top panels show grand averaged relative power maps, with color bars scaled to the maximum values of the map, with alpha (A) showing predominantly occipital power and beta (D) showing high power in the sensorimotor cortices. Middle panels display relative power extracted per participant, with one plot for each lobe. Each line represents a participant, with visit on the x axis and the average relative power across each lobe on the y axis. ICC estimates are inset and show that the highest estimates were in the parietal lobe for alpha (B) and beta (D). Bottom panels show vertex-wise ICC estimates for the three visits, scaled from 0 to 1. Alpha (C) remains relatively reliable across the cortex, while beta (F) shows high estimates posteriorly, which progressively decreases moving anteriorly.

Grand average maps of resting alpha (9 −12 Hz) power showed the highest power in the occipital cortex, with power generally decreasing anteriorly across the cortex (Fig. 2A). ICC estimates at each of the lobes broadly revealed moderate to good levels of reliability, with the highest estimates in the parietal cortex (ICC = .785, 95% CI: .598-.905), followed by the frontal lobe (ICC = .748, 95% CI: .538-.887), temporal lobe (ICC = .687, 95% CI: .455-.855), and the qualitatively lowest reliability within the occipital lobe (ICC = .658, 95% CI: .414-.840 Fig. 2B). Vertex-wise ICC maps showed generally stable reliability across the cortex, with the highest levels near the sensorimotor and parietal cortices (Fig. 2C). Overall, few areas across the cortex fell below an ICC of 0.5 (poor reliability).

### Beta

Grand average maps of resting beta (15–30 Hz) power showed the highest power in the sensorimotor cortices, with relatively high power extending through the premotor areas (Fig. 2D). ICC estimates at each of the lobes showed the highest estimates at the parietal cortex (ICC = .853, 95% CI: .714-.937), followed by the occipital lobe (ICC = .782, 95% CI: .595-.903), temporal lobe (ICC = .681, 95% CI: .439-.853), and the lowest reliability within the frontal lobe (ICC = .646, 95% CI: .400-.833; Fig. 2E). Notably, beta power in the parietal lobe showed the highest ICC estimates across all lobes and frequency bands. Vertex-wise ICC maps showed a large cluster of good reliability extending across the parietal and occipital cortices (Fig. 2F). ICC estimates decreased anteriorly across the cortex, with poor values seen in the anterior prefrontal cortices and temporal poles.

### Gamma

Grand average maps of resting low gamma (30–80 Hz) and high gamma (80–150 Hz) power showed similar distributions of relative power, with the highest power in the anterior prefrontal cortices (Fig.s 3A & 3D). ICC estimates at each of the lobes displayed broadly poor reliability, with the highest estimates in the occipital cortex (Low Gamma: ICC = .639, 95% CI: .277 .846; High Gamma: ICC = .530, 95% CI: .157-.787), followed by the parietal lobe (Low Gamma: ICC = .537, 95% CI: .142-.795; High Gamma: ICC = .345, 95% CI: .044-.645), temporal lobe (Low Gamma: ICC = .509, 95% CI: .104-.782; High Gamma: ICC = .371, 95% CI: .046-.673), and the lowest reliability in the frontal lobe (Low Gamma: ICC = .262, 95% CI: .000-.568; High Gamma: ICC = .067, 95% CI: −.064-.299; Fig.s 3B & 3E). Vertex-wise ICC maps showed a small spatial cluster of moderate reliability in the lateral occipital (Fig.s 3C & 3F). Overall however, ICC estimates for both gamma bands showed the lowest ICCs of all of the frequency bands, with most of the cortex below 0.5, and many areas even approaching 0.

## Discussion

In the current study, we examined the three-year test-retest reliability of resting-state power as measured by MEG using the ICC. Overall, our results showed that alpha and beta power had the highest reliability estimates, while gamma power had the lowest estimates of three-year reliability. Spatially, the parietal cortex appeared to show the highest degree of reliability across multiple frequencies, including delta, alpha, and beta. These findings provide critical insight into the stability of frequency- and spatially-specific resting-state power and help guide future MEG investigations across multiple fields.

Our results replicate multiple findings from the (Martín-Buro et al., 2016) study of resting-state reliability in MEG, although with some no-table differences. In a sample of young adults, they identified high reliability over one week in temporal theta, fronto-posterior alpha, and frontal low beta power. While we also noted high reliability for alpha and beta, theta power in our sample was not as reliable, and instead, delta power showed higher degrees of reliability. Spatially, we generally found higher reliability more posteriorly, specifically in the parietal cortex. Beyond these differences in findings, this study expands upon previously reported data as the first report on the long-term reliability of resting-state MEG oscillatory activity.

Additionally, our sample is novel in that our participants spanned a range of ages across adulthood. Many neuroimaging studies that measured reliability have examined a limited sample of young adults, which may or may not generalize to older adults. At the same time, our research focused on the maximum range of adulthood without including the potential extremes of development (*<* 24 years) or aging (65 +). Assessing reliability across long periods of time and throughout normal adulthood is critical in establishing the stability and robustness of these measurements, and ultimately to help move these metrics forward as biomarkers of disease. This is especially the case when such metrics are used to monitor the progression of diseases over time. In this case, oscillatory power explicitly measured during the resting state is also efficient for biomarker identification and development. Resting-state protocols can be easily standardized and applied to patient populations that may have trouble completing more complex paradigms (e.g., those with Alzheimer’s disease).

In our study, alpha and beta power showed the highest ICC estimates overall. This aligns with previous studies, mainly showing high stability of resting-state alpha band measures (Gasser et al., 1985; Näpflin et al., 2007). Alpha and beta are the two dominant rhythms in the brain and therefore were expected high-reliability estimates for these spectral bands. While the functional role of alpha and beta activity is highly dependent on location in the cortex and cognitive task, alpha activity is broadly thought to reflect active inhibitory control (Klimesch et al., 2007), particularly of activity related to visual and attentional processing (Klimesch, 2012). Beta activity is generally associated with sensorimotor and cognitive control (Engel and Fries, 2010). As expected, we, therefore, found the highest relative alpha power in the occipital cortex and the highest relative beta power in the sensorimotor cortices.

Interestingly, however, these locations were not where the highest ICC estimates were found for each respective frequency band. Beta power showed higher ICC estimates in the occipital cortex than alpha power. Additionally, parietal beta power had the highest ICC, despite relative beta power in the parietal cortex being weaker than parietal power at all other frequency bands other than gamma. Interestingly, across all frequency bands, ICC maps did not reflect similar spatial distributions to the grand-averaged power maps. This suggests that the reliability of resting-state MEG estimates is not solely dependent on the signal to noise ratio. That said, high gamma power did show the lowest overall ICC and had the lowest power. This may suggest that resting-state gamma power has poor reliability due to poor signal to noise. Task-based paradigms that induce strong gamma responses may elevate gamma signal and show better reliability, as seen in previous studies (H.-R. Tan et al., 2016; Tan et al., 2015). Caution may be warranted, however, when interpreting resting gamma activity, particularly beyond the occipital cortex.

Spatially, higher ICC estimates in the parietal cortex relative to other cortical regions may be related to the acquisition method. These measurements were acquired with a 306-sensor Elekta/MEGIN MEG system, which contains a traditional SQUID sensor array. Given the layout of the fixed helmet, participants may systematically be seated such that the parietal cortex lies the closest to the sensor array. Indeed a recent study has shown that fixed-helmet cryogenic systems are most sensitive to measurements from the parietal cortices (Hill et al., 2020). This could be one explanation for why parietal areas seemed to show higher ICC estimates across multiple frequency bands. That said, being closest to the sensor array most strongly affects signal strength, and our results show that our estimates of stability was not tightly coupled to MEG signal strength across multiple bands. With the advent of optically-pumped magnetometer systems, which can use flexible sensor arrays (Hill et al., 2020), further study will be possible to dissociate this. Further, such systems may achieve enhanced reliability, particularly beyond the parietal cortices.

Given this study’s focus on the reliability of neuroimaging measures, we would like to highlight our use of an established and open-access analysis pipeline (Niso et al., 2019). This is critical for good scientific practice, as we show that the results of the pipeline are reproducible, and further establish which specific metrics that result from the pipeline are reliable. We also show that inter-rater reliability of this pipeline yields excellent ICC values (Fig. S1). Variability in neuroimaging has long been attributed to differences and flexibility in analysis pipelines (Botvinik-Nezer et al., 2020). Therefore, future studies must continue to study and compare other analysis pipelines, with the hope that further standardization of analyses can decrease methodological variability across studies.

Before closing, it is important to note the limitations of our study. First, it is limited in that it focuses on a group of healthy adults. The test-retest reliability of oscillatory activity in pediatric populations may be very different, and it is widely known that such frequency-specific activity does change with development (Fung et al., 2020; Heinrichs-Graham et al., 2020; Taylor et al., 2021; Uhlhaas et al., 2010; Whitford et al., 2007). Our sample also did not extend into older adult-hood, where either normative or pathologic aging may significantly alter the stability of these measurements (Arif et al., 2020; Heinrichs-Graham et al., 2018; Spooner et al., 2019; Wiesman and Wilson, 2019). Additionally, long-term stability of activity may not generalize to clinical populations. Concerning those frequencies and regions of the brain found to have lower ICC measures of reliability, it is difficult to distinguish whether low ICC estimates were due to measurement inaccuracy (as is often concluded in short-term test-retest reliability studies) or true dynamic change in power over the three years. Our study likely highlights stable neurophysiologic traits, and because of the long-term nature of the study, measurements with low ICC values may be due to a relationship with a neurophysiologic state that may have been more variable from visit to visit. Related to this specifically, we noted unexpectedly stronger broadband gamma power during visit 1 relative to visit 2 and 3 across many regions. Given the trajectory of this and previous studies showing increasing gamma power with age (Hunt et al., 2019), we suspect this could be related to physiological noise related to muscle artifact secondary to anxiety, which participants may have exhibited less often during visits 2–3 because they were acclimated to the environment. Further study is therefore needed to examine such change over time versus reliability of resting theta and gamma power. That said, our findings provide added confidence on measures of alpha and beta power in such healthy control samples.

In summary, our three-year longitudinal study of MEG responses showed high degrees of stability in parietal delta, posterior beta, and alpha across most of the cortex, with less stability in estimates of gamma power. Thus, this study helps establish oscillatory activity as stable across an appreciable period, opening up the use of these responses in further longitudinal studies and supporting their use for comparisons to patient populations in hopes of identifying potential biomarkers.

## Supplementary Material

1

## Figures and Tables

**Fig. 3.**
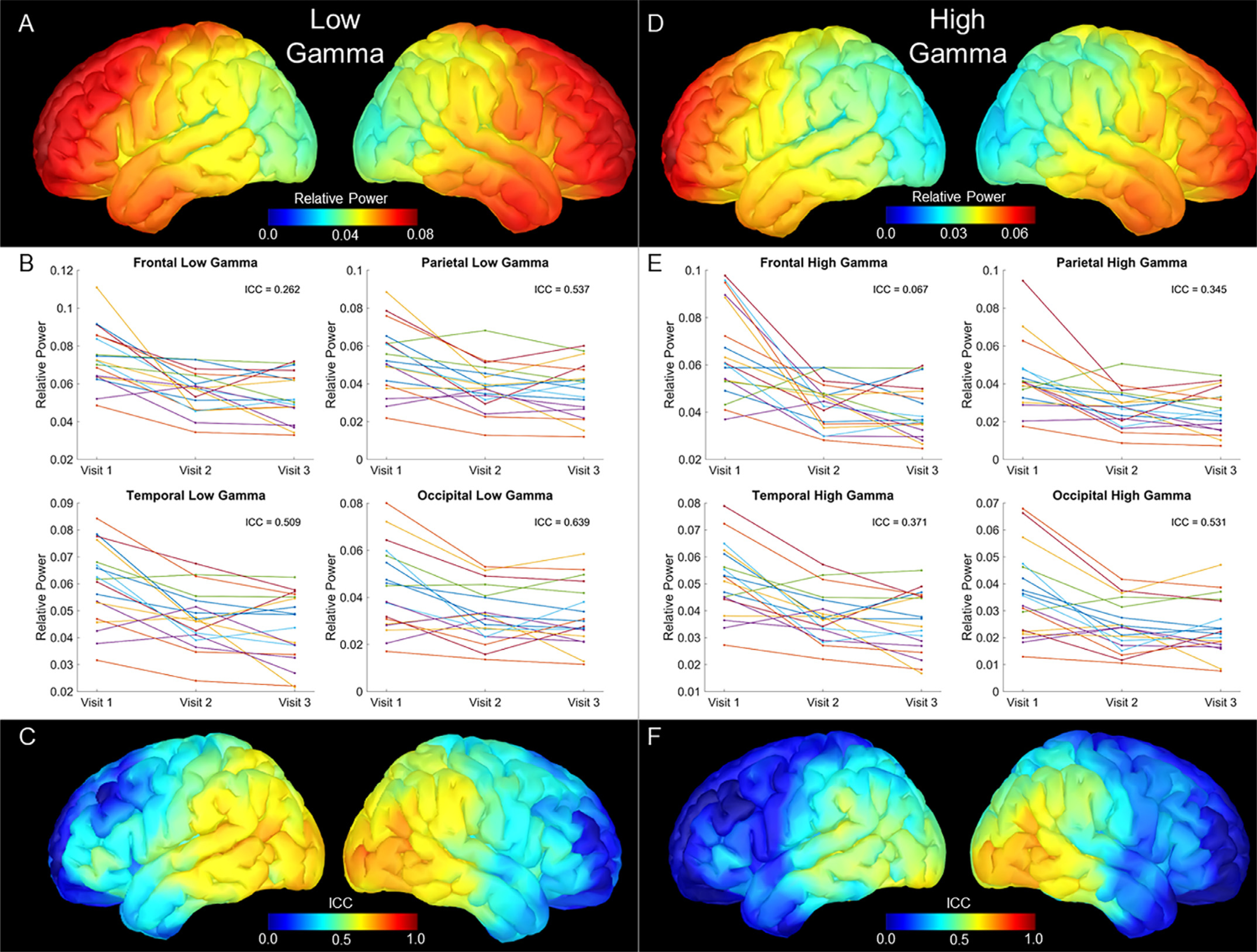
Low and High Gamma Relative Power Estimations and 3-year ICC Estimates. Top panels show grand averaged relative power maps, with color bars scaled to the maximum values of the map, with low gamma (A) and high gamma (D) showing predominantly frontal power. Middle panels display relative power extracted per participant, with one plot for each lobe. Each line represents a participant, with visit on the x axis and the average relative power across each lobe on the y axis. ICC estimates are inset and show the highest estimates were in the occipital lobe for both low gamma (B) and high gamma (D). Bottom panels display vertex-wise ICC estimates for the three visits, scaled from 0 to 1. Low gamma (C) showed broadly poor reliability, with some moderate levels restricted to the lateral occipital cortices, while high gamma (F) displayed a similar topography with even lower ICC estimates.

## Data Availability

The data used in this article can be made available under reasonable request. Data processing pipelines followed previous studies (Niso et al., 2019) using a combination of Brainstorm (Tadel et al., 2011), which is documented and freely available for download online under the GNU general public license (http://neuroimage.usc.edu/brainstorm), and CAT12 (Gaser and Dahnke, 2016) toolboxes.
