# Supplementary material for: Three-year reliability of MEG resting-state oscillatory power
Source: Neuroimage. Author manuscript; Available in PMC 2021 Nov 14. (PMC8590732; doi:10.1016/j.neuroimage.2021.118516)
Supplement: 1 [file NIHMS1748164-supplement-1.docx]

Supplement


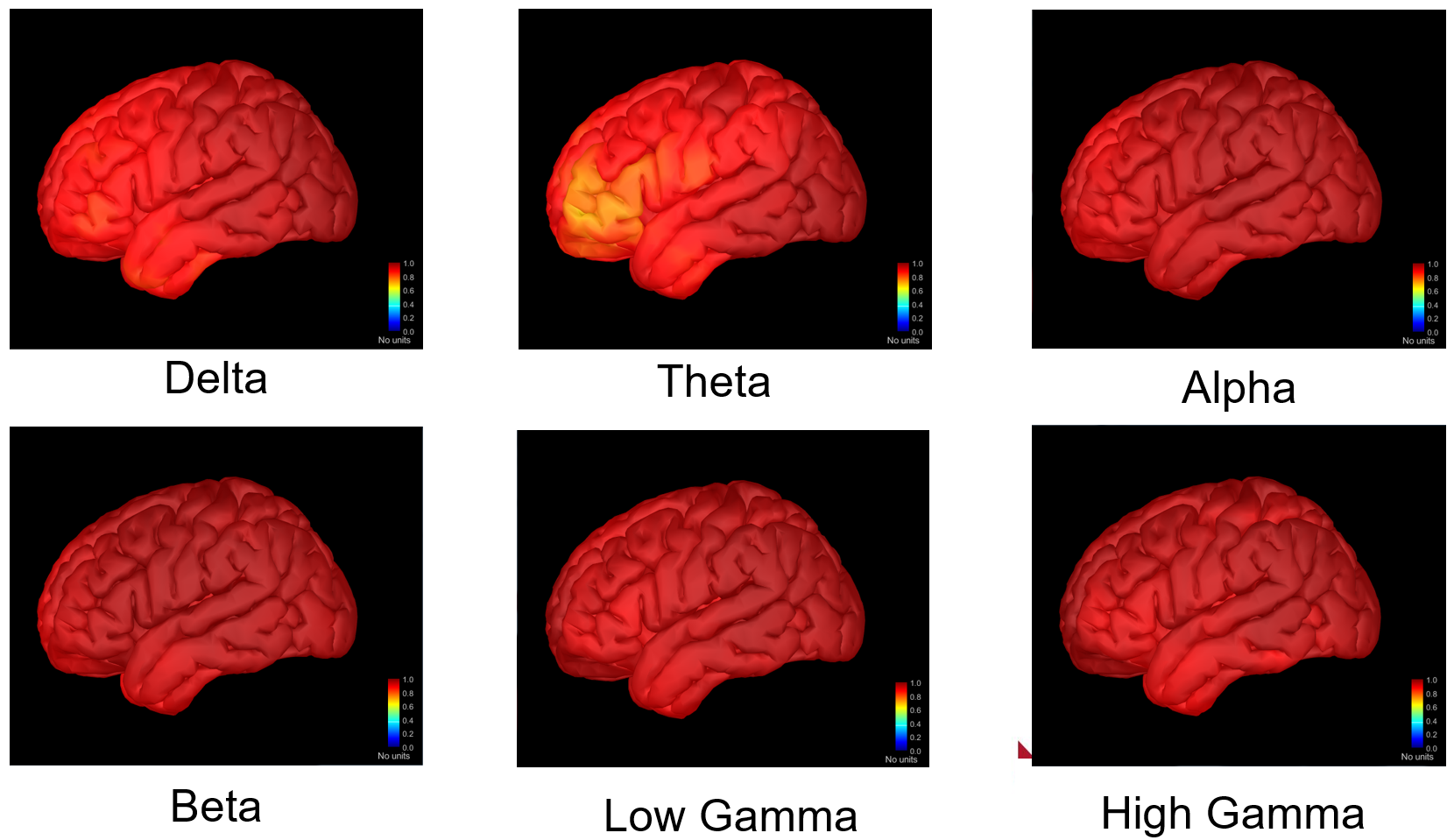


Figure S1: Inter-rater Reliability of the Resting State Pipeline. Three investigators separately analyzed the entire dataset and the source-level power spectral density maps were compared using intra-class correlation (ICC). All maps showed excellent inter-rater reliability, ultimately displaying the consistency of implementation of the resting state analysis pipeline. All maps are display inter-rater ICC values scaled from 0 (blue) to 1 (red) for each respective frequency band.
